# Heterotrophic feeding and symbiotic state shape the chemical microenvironment, microbiome composition, and activity in the coral gastrovascular cavity

**DOI:** 10.1128/aem.02441-25

**Published:** 2026-07-27

**Authors:** Qingfeng Zhang, Elena Bollati, Michael Kühl

**Affiliations:** 1 Marine Biology Section, Department of Biology, University of Copenhagen 453226, Helsingør, Denmark; 2 School of Life Sciences, University of Essex 2591, Colchester, United Kingdom; Universidad de los Andes, Bogotá, Colombia

**Keywords:** coral microbiome, holobiont, microbial activity, microenvironment, symbiosis, gastrovascular cavity

## Abstract

Coral gastrovascular cavities (GVCs) host diverse microorganisms and, as semi-closed compartments, are characterized by elevated nutrient concentrations and pronounced diel fluctuations in oxygen (O_2_) and pH. However, their broader chemical microenvironment and associated microbial activities remain poorly understood. Here, we provide direct evidence for active anaerobic metabolism in the GVC of *Astraeosmilia curvata* by measuring hydrogen (H_2_), nitric oxide (NO), and nitrous oxide (N_2_O) production, indicative of microbial fermentation and denitrification. Changes in GVC H_2_ concentrations in *Astraeosmilia curvata* and *Goniastrea* sp. after food particle ingestion suggest that coral feeding activity modulates substrate availability, which in turn influences microbial activity in this compartment. In bleached *A. curvata* corals, the GVC remained consistently hypoxic and acidic, and anaerobic metabolic processes persisted across the diel cycle, whereas in healthy corals, the GVC became hyperoxic under light, and anaerobic metabolism was suppressed. These patterns suggest a shift in energy and nutrient cycling pathways within the bleached coral holobiont. Amplicon sequencing and PICRUSt2-based functional predictions revealed that the shifts in bacterial community composition correlated with microenvironmental chemical gradients, including the enrichment of putative denitrifying taxa (Alcanivoracaceae, Xanthobacteraceae) in polyps exhibiting elevated NO concentration, alongside predicted metabolic pathways involved in nitrogen and hydrogen metabolism. Together, our findings identify the GVC as a dynamic site of microbial activity that may play an important role in holobiont carbon and nitrogen cycling, with potential implications for nutrient balance and coral resilience.

**IMPORTANCE:**

Understanding interactions among the coral animal host, Symbiodiniaceae, and associated bacteria within specific coral compartments is essential for elucidating the underlying mechanisms affecting the ecophysiology of reef-building coral holobionts, both under ambient and environmental stress conditions. However, most studies in the literature have focused on studying coral-microbe interactions at larger spatial scales, and especially the gastrovascular cavity (GVC) of corals remains underexplored despite its key role in coral processes, such as prey digestion, circulation of resources between polyps, sexual reproduction, and as the pathway for algal symbiont expulsion and colonization. By combining microsensor profiling with microvolume sampling and molecular analysis of the gastrovascular cavity, this study demonstrates strong chemical dynamics in single coral polyps, where spatio-temporal changes in O_2_ availability enable active fermentation and denitrification within the GVC. Our findings suggest that the GVC is a hotspot of both aerobic and anaerobic microbial metabolism, with potential importance for energy and nutrient cycling in the coral holobiont.

## INTRODUCTION

Tropical, reef-building corals represent a multispecies assemblage of calcifying cnidarian polyp animals, their microalgal endosymbionts (in the dinoflagellate family Symbiodiniaceae), as well as multiple microbiomes associated with the animal tissue surface, the animal gastrovascular cavity (GVC), and the underlying coral skeleton forming the coral colony scaffold (1, 2). While the microenvironmental conditions in these compartments of the coral holobiont can be very different (3–7), most ecophysiological studies of corals are done at the level of whole coral colony fragments. Likewise, there is increasing evidence for differential composition of microbiomes in different coral holobiont compartments (8–11), and there is a rising awareness among coral biologists of the need for higher spatial resolution studies to unravel functional links between the coral host and different microbial communities in the holobiont (12).

The Symbiodiniaceae within coral tissues not only shape the O_2_ and pH microenvironment of corals via the interplay of symbiont photosynthesis, host and symbiont respiration (3), but also fundamentally determine the coral holobiont’s energy acquisition (13). Upon coral bleaching, i.e., the breakdown of this mutualistic symbiosis due to environmental stressors, the loss of algal symbionts disrupts the primary energy supply of the host and forces corals to shift their energy acquisition toward heterotrophy (14–16). Such changes may also profoundly alter the nutrient composition and redox state within the GVC. Consequently, its microbial community composition and activity may also be altered, potentially affecting holobiont recovery and survival. Yet, how different compartments and associated microbes respond to bleaching remains poorly understood, leaving a critical gap in our knowledge of coral holobiont function under stress.

The GVC of corals plays a central role in their biology, e.g., as a site for food digestion, nutrient uptake, and sexual reproduction, as a pathway for departing or arriving symbionts during coral bleaching and recovery, as well as for resource sharing between neighboring polyps (1, 7, 17). The semi-enclosed nature, small volume, and large internal tissue surface area of the GVC lead to strong spatio-temporal variations in O_2_ concentration and pH depth gradients in the GVC between light and dark conditions affecting, e.g., the internal carbonate chemistry and calcification process in corals (5, 18–23). Furthermore, corals acquire organic matter through heterotrophic feeding, and the GVC can contain up to 10–100-fold higher concentrations of dissolved inorganic nitrogen species (including nitrate, nitrite, and ammonium) than the surrounding seawater (5). These inorganic nitrogen compounds serve as important substrates for microbial metabolism, with nitrate and nitrite functioning as electron acceptors for anaerobic respiratory processes, such as heterotrophic denitrification, while ammonium can stimulate nitrification in the presence of O_2_.

While the chemical conditions in the GVC of some corals switch from more uniform high pH and hyperoxia in light to low pH and hypoxia/anoxia in darkness, other species with deeper GVCs, such as *Galaxea fascicularis*, *Dipsastraea favus*, and *Lobophyllia hemprichii*, can exhibit strong stratification of their chemical microenvironment, rendering deeper parts of the GVC hypoxic or anoxic even in light (5, 23, 24). Such spatiotemporal dynamics potentially allow for the presence of anaerobic or microaerophilic microbes in the coral GVC; however, so far, direct evidence of anaerobic processes in the GVC via microsensor measurements of, e.g., H_2_ (indicative of fermentation or N_2_ fixation), NO or N_2_O (indicative of denitrification), or H_2_S (indicative of sulfate reduction) is lacking.

In this study, we investigated the microenvironmental dynamics of the GVC. Specifically, we examined how the coral’s symbiotic state and heterotrophic feeding influence the chemical microenvironment within the GVC, and whether variations in these chemical gradients are associated with shifts in bacterial community composition and microbial activities. To address these questions, we combined microsensor measurements with microvolume sampling and 16S rRNA gene metabarcoding of healthy and bleached polyps of *Astraeosmilia curvata*, and further assessed H_2_ dynamics following feeding in *A. curvata* and *Goniastrea* sp. Our study represents the hitherto most detailed characterization of the GVC microenvironment and its implications for microbial diversity in this central compartment of the coral holobiont.

## MATERIALS AND METHODS

### Coral husbandry

Colonies of *Astraeosmilia curvata* were obtained from DeJong Marine Life (the Netherlands) and maintained in a recirculating aquarium system at the Marine Biology Section, University of Copenhagen (Helsingør, Denmark). Upon delivery, three polyps appeared visibly bleached (possibly due to low-temperature stress during transport). Healthy and bleached corals were kept under controlled conditions: temperature 25°C, salinity 35, and photon irradiance (400–700 nm) of 150 μmol photons m^−2^ s^−1^, with a 12 h light:12 h dark cycle for at least 7 days before measurement. The system maintains nitrate concentration below 1 mg L^−1^ and phosphate concentration between 0.009 and 0.016 mg L^−1^. The same sets of healthy *A. curvata* polyps (polyps 1–3; biological replicates) and bleached *A. curvata* polyps (polyps 1–3; biological replicates) were used for O_2_, pH, H_2_, NO, and GVC microsample analyses. For N_2_O measurements, a separate set of healthy *A. curvata* polyps from a different colony was used. Two fragments of *Goniastrea* sp. were collected from the reef flat of Heron Island (Great Barrier Reef, Australia; permit no. G24/49877.1) in May 2024 using a hammer and chisel, and kept at an outdoor flow-through aquarium flushed with seawater from the reef for 7 days before measurement.

### Microsensor measurements

Microsensors for O_2_, pH, H_2_, H_2_S, NO, and N_2_O (Unisense A/S, Denmark) with a tip diameter of 50 μm were used to measure chemical dynamics in the coral GVC microenvironment. The microsensors (and a reference electrode for pH measurements; Ag/AgCl, Unisense A/S, Denmark) were connected to a multichannel microsensor meter (fx-6 UniAmp, Unisense A/S, Denmark) and mounted on a motorized micromanipulator (Unisense A/S, Denmark) that enabled precise vertical movement at μm-scale resolution. The microsensor meter and micromanipulator were connected to a PC with dedicated software for data acquisition and sensor positioning (SensorSuite Profiler v3.4, Unisense A/S, Denmark). Each microsensor was calibrated at the experimental temperature and salinity prior to measurement.

The O_2_ microsensors were linearly calibrated from sensor signal readings (in pA) using a two-point method in 100% air-saturated seawater and anoxic water (using a sodium ascorbate solution). The pH microsensors were calibrated from sensor readings (in mV relative to the reference electrode) in standard buffer solutions of pH 4.0, 7.0, and 10.0 (Hach, USA). The H_2_ microsensors were linearly calibrated from sensor readings (in pA) in seawater from the experimental aquarium (0 μM H_2_) and seawater flushed with a gas mixture of 5% H_2_ and 95% N_2_ gas, yielding an H_2_ concentration of 33.4 μM at experimental temperature and salinity according to tabulated values of gas solubility in water (Unisense). The NO microsensor was calibrated using a solution containing 0.1 M H_2_SO_4_ and 0.1 M KI, which was flushed with N_2_ gas to remove dissolved O_2_. 200 μL of 1 mM NaNO_2_ solution was added stepwise into the 200 mL calibration solution. Each addition generated 1 μM NO, and the NO microsensors were linearly calibrated from microsensor readings (in pA) in 0, 1, 2, and 3 μM NO solutions. The N_2_O microsensor was linearly calibrated from readings (in pA) in seawater from the experimental aquarium (0 μM N_2_O) and a 100 μM N_2_O solution prepared by adding 0.95 mL of N_2_O-saturated solution to 199.05 mL seawater. The H_2_S microsensor was linearly calibrated from readings (in pA) using a pH 4 buffer solution (salinity 35) flushed with N_2_ gas to remove oxygen. The 0 µM solution was the buffer, a 100 µM H_2_S solution was prepared by adding 0.1 mL of 0.01 M Na_2_S to 9.9 mL buffer, and a 50 µM H_2_S solution was obtained by mixing equal volumes of the 0 and 100 µM solutions.

The positioning of the microsensor tip relative to the coral mouth was monitored using a digital microscope (Dinolite, AnMo Electronics, Taiwan). The sensor tip was manually aligned to the center of the coral mouth and was then lowered in 20–50 μm steps using the motorized micromanipulator until coral tissue contraction was observed, which was defined as the bottom of the GVC. Measurements were initiated after the sensor signal had stabilized for 5 min.

During microsensor measurements, the coral fragment was placed in a custom-designed flow chamber with a constant laminar flow (1 cm s⁻¹) of thermostated and oxygenated seawater (kept at 25°C and a salinity of 35) supplied from a supporting aquarium, as previously described by Brodersen et al. and Zhang et al. (25, 26). During light measurements, the coral fragment was illuminated by a white LED lamp (KL 2500 LED, Schott, Germany) providing a photon irradiance (400–700 nm) of 100 μmol photons m^−2^ s^−1^ (low light) or 200 μmol photons m^−2^ s^−1^ (high light). Photon irradiance was quantified with a photon irradiance meter (ULM-500, Heinz Walz, Germany) equipped with a spherical micro quantum sensor (US-SQS/L, Heinz Walz, Germany). For H_2_ and NO measurements on healthy polyps, high-light measurements were only conducted if H₂ or NO was detected under low-light conditions.

For feeding experiments, coral fragments were fed with coral food (ReefPearls 5–200 µm, DVH Aquatic, the Netherlands) 20–24 h prior to measurements. In this study, heterotrophic feeding refers specifically to the ingestion of food particles by the coral. For H_2_ monitoring after food particle ingestion, *A. curvata* was kept under a 12 h:12 h light–dark cycle for 36–48 h, and measurements were conducted at three time points after food particles were ingested by the coral polyps. For continuous H_2_ measurements in the GVC of field-collected *Goniastrea* sp. (see setup details below), coral food was delivered directly to the oral disks of target polyps using a pipette; the microsensor tip was positioned at approximately 50% of the cavity depth, and measurements were initiated immediately after the coral polyps ingested the food particles.

Microsensor data in the GVC were averaged over the portion of each profile with depths ≤0, and mean values and standard deviations were then calculated across different light conditions, coral symbiotic states, and time points following food capture. Differences between healthy and bleached corals were assessed using independent Welch’s *t*-tests. The effect of light conditions on the GVC chemical microenvironment was evaluated with one-way ANOVA, followed by Tukey’s HSD post hoc tests for pairwise comparisons. All analyses were performed using the *dplyr* and *stats* packages in R. For data visualization, we used ggplot2 in R to plot individual replicate profiles as dashed lines, with LOESS-smoothed trends and 95% confidence intervals shown as continuous lines with gray shading (27).

### Microvolume sample collection, DNA extraction, and 16S rRNA gene metabarcoding

For amplicon sequencing-based microbiome analysis, GVC fluid samples were collected after microsensor measurements. During sampling, coral fragments were placed in a glass container that had been rinsed with 70% ethanol and filled with 0.22 μm-filtered seawater to avoid cross-contamination. Microvolume samples of GVC fluid were collected from *A. curvata* polyps using a sterile 1 mL syringe equipped with a low dead-space microneedle (34G, TSK, Canada) as described in Bollati et al. (24). Seawater samples (100 µL) were also collected from the sampling container, aquarium tank, and flow chamber using the same method. All samples were transferred into UV-crosslinked (1 h) 1.5 mL sterile microcentrifuge tubes and stored at −70°C until processing.

DNA extraction was performed in a UV-sterilized hood using a low-input (10 µL) physical lysis protocol, following Bramucci et al. and Bollati et al. (24, 28). The V3–V4 hypervariable region of the 16S rRNA gene was amplified using Illumina fusion primers (341 F: TCGTCGGCAGCGTCAGATGTGTATAAGAGACAGCCTAYGGGRBGCASCAG and 805 R: GTCTCGTGGGCTCGGAGATGTGTATAAGAGACAGGGACTACNNGGGTATCTAAT). Each 25 µL PCR reaction contained 5 µL template DNA, 12.5 µL KAPA HiFi HotStart ReadyMix (Roche, Switzerland), 0.5 µL of each 10 µM primer, and 6.5 µL PCR water. PCR amplification was performed with an initial denaturation at 98°C for 2 min, followed by 35 cycles of 98°C for 30 s, 55°C for 30 s, and 72°C for 30 s, with a final elongation at 72°C for 10 min. Amplicons were visualized by agarose gel electrophoresis and sent to the Australian Genome Research Facility (Melbourne, Australia) for library preparation and sequencing on the Illumina NextSeq platform (300 bp paired-end). Two extraction negatives were included and sequenced alongside the samples.

### Sequencing data processing

Demultiplexed sequences were trimmed to remove adapter and primer sequences using cutadapt (v4.4) (29) and subsequently processed in R (v4.1.1) with the DADA2 pipeline (v1.22) (30). Forward and reverse reads were truncated at 250 bp, and the maximum number of expected errors was set to 2. Filtered reads were denoised, merged, and screened for chimeras to infer amplicon sequence variants (ASVs). Taxonomic classification of ASVs was performed against the SILVA reference database (v138.1) (31). ASVs in extraction blanks and ASVs identified as eukaryotic, chloroplast, or mitochondrial sequences were removed. Rarefaction curves were generated using the vegan package (v2.7-1) (32) to verify sufficient sequencing depth, and no rarefaction was applied.

Alpha diversity was calculated from the ASV table in *phyloseq* v1.52 as Shannon’s H index and visualized as its exponential (eH). Differences in alpha diversity among SampleType groups were compared using the Kruskal–Wallis test, followed by pairwise Wilcoxon rank-sum tests with Benjamini–Hochberg correction. Beta diversity was assessed using Bray–Curtis dissimilarity, and microbial community composition was visualized via principal coordinates analysis (PCoA) using *phyloseq*. Differences in community composition were tested via PERMANOVA (adonis2, 999 permutations), and homogeneity of dispersion was confirmed using *betadisper* with ANOVA in the R package vegan v.2.7-1 (32). Differential abundance was evaluated pairwise using ALDEx2 v1.26.0 (33) for taxa aggregated at the family level to identify families exhibiting significant differences in relative abundance across sample groups. Comparisons were performed across groups defined by (i) coral symbiotic state (healthy vs bleached polyps) and (ii) microenvironmental conditions based on NO and H_2_ concentrations measured by microsensors. Polyps exhibiting very low (near-zero) NO or H_2_ concentrations in the dark were selected and compared to other samples. Taxa were considered significant if they had an unadjusted *P* value < 0.05 and the 95% confidence interval of effect size overlap was less than 20%.

Functional prediction of microbial communities was performed using PICRUSt2 (v2.5.0) (34) to generate KEGG Ortholog (KO) functional profiles. Subsequent analyses focused on anaerobic metabolic pathways, including denitrification, nitrogen fixation, hydrogen production and consumption, sulfate reduction, and sulfide oxidation pathways (Table S1). ASVs corresponding to target KOs were identified, and the predicted abundance of each function was calculated as the relative abundance of ASVs assigned to any of the corresponding KOs in each sample. The predicted abundance of selected functional genes was compared between groups using the Wilcoxon rank-sum test, including the antioxidant enzyme catalase between healthy and bleached corals; while predicted denitrification and hydrogen metabolism functions were compared among groups defined by different NO and H_2_ concentrations measured by microsensors. All analyses were performed in R v4.4.3.

## RESULTS

### Chemical dynamics in the coral GVC

**Fig 1:**
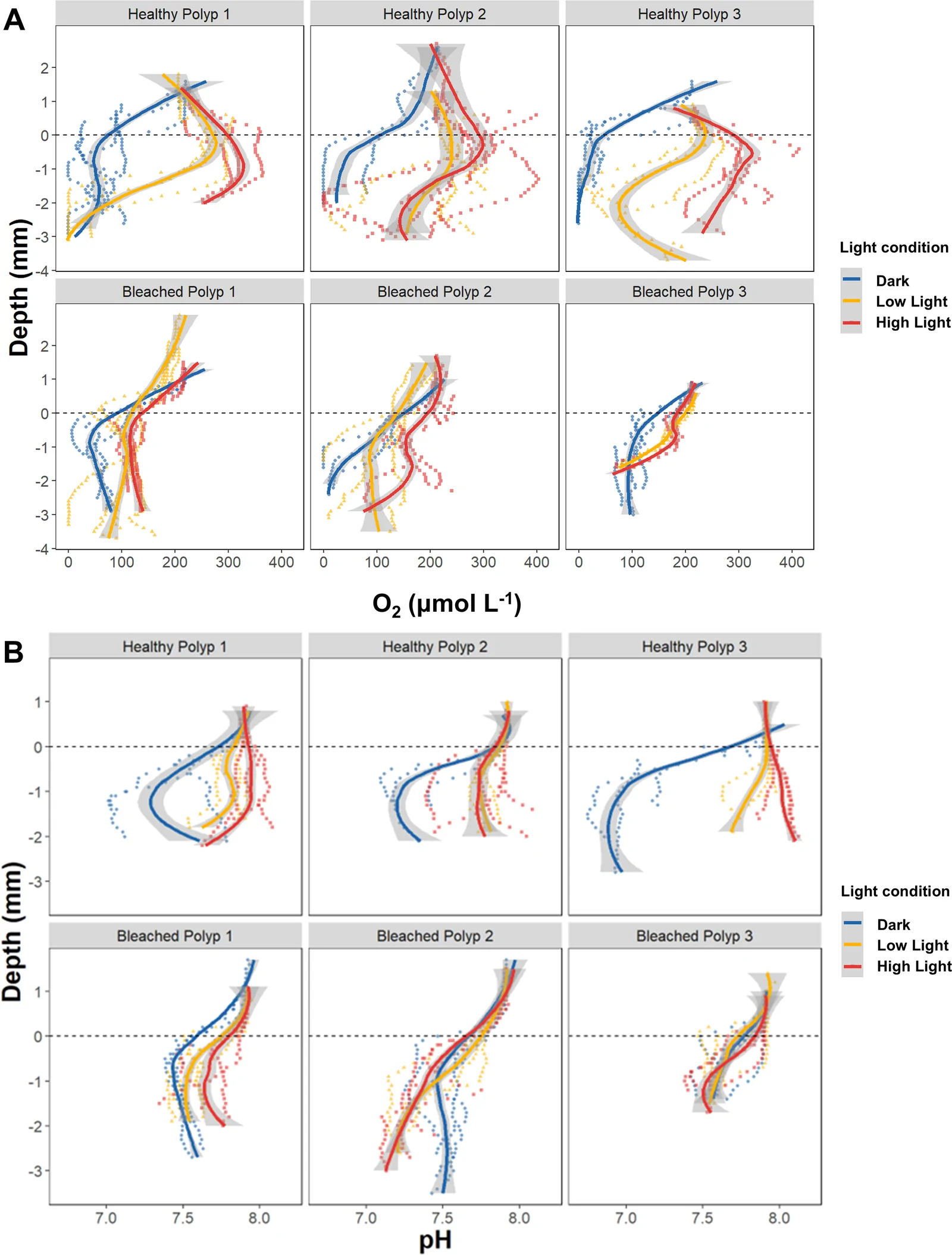
Depth profiles of O_2_ concentration (**A**) and pH (**B**) in the gastrovascular cavity of healthy and bleached *A. curvata* polyps in darkness and under an incident photon irradiance (400–700 nm) of 100 µmol photons m^−2^ s^−1^ and 200 µmol photons m^−2^ s^−1^. The dashed lines indicate replicate measurements (three technical replicates per polyp), while the continuous lines indicate LOESS-smoothed trends with 95% confidence intervals (gray shading). Zero depth represents the level of the coral mouth opening, while negative depth values indicate positions inside the GVC.

In the GVC of healthy *A. curvata*, O_2_ concentrations reached 350–400 μmol O_2_ L^−1^ under high light, 250–300 μmol O_2_ L^−1^ under low light, and dropped to below 100 μmol O_2_ L^−1^ in the dark (Fig. 1A). Spatial profiling revealed that under light conditions, O_2_ levels were higher in the upper region of the GVC and then decreased with depth, while O_2_ concentration declined from the mouth opening into the GVC during darkness. However, technical replicate measurements on the same coral polyp also showed pronounced fluctuations under constant light conditions. For example, in Healthy Polyp 2, the O_2_ concentration shortly dropped to anoxic level under high light. In comparison, bleached corals showed consistently lower O_2_ levels (below 200 μmol O_2_ L^−1^, Fig. 1A; Table S2), with only slight increases under light relative to dark conditions.

Similar to O₂ dynamics, pH variations were mainly governed by the light-dark cycle. In the healthy corals, pH in the GVC varied by >1 pH unit, i.e., from <pH 7.0 in the dark to >pH 8.0 under high light. In bleached corals, pH remained consistently lower than ambient seawater pH under both dark and light conditions (Fig. 1B).

**Fig 2:**
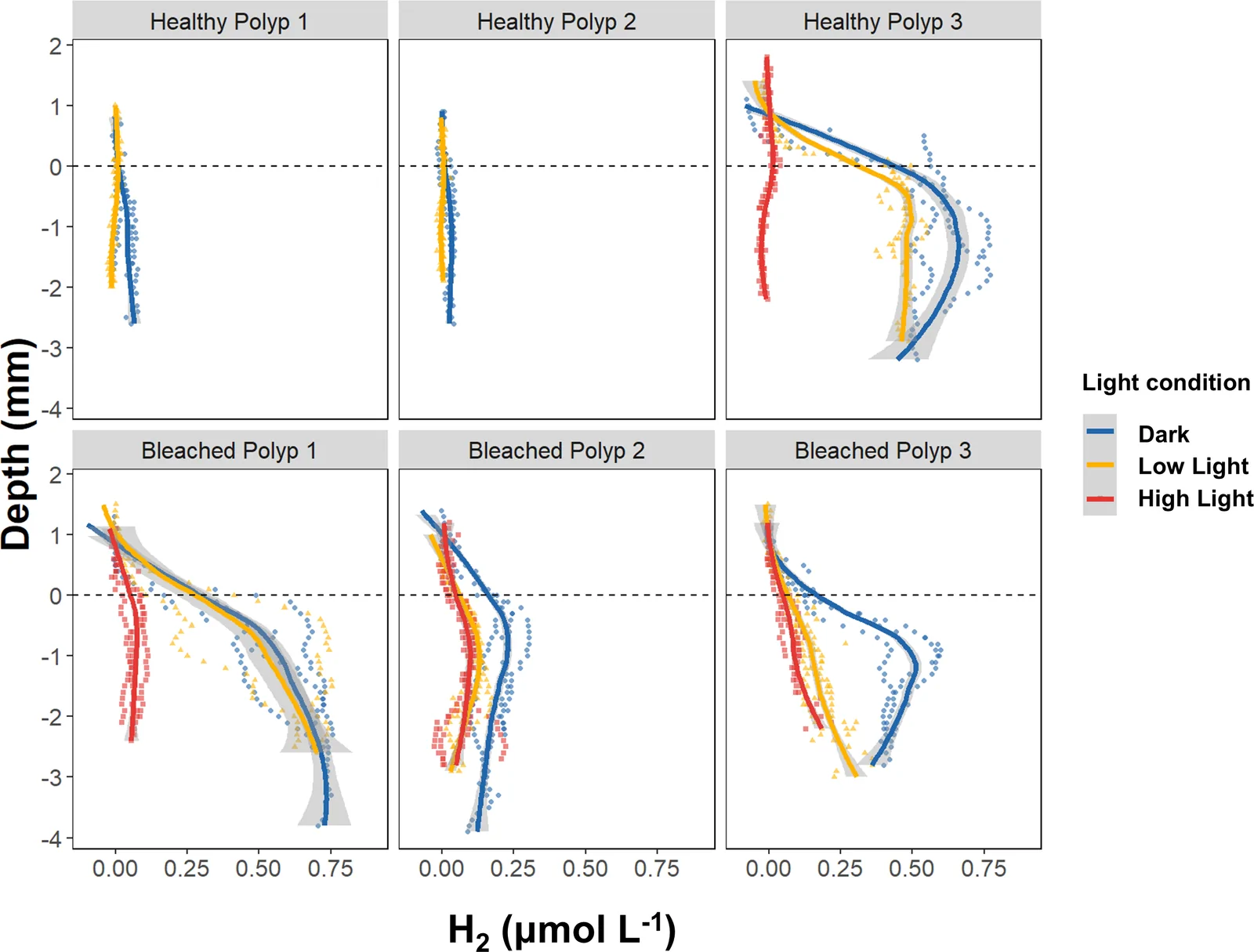
Depth profiles of H_2_ concentration in the gastrovascular cavity of healthy and bleached *A. curvata* polyps in darkness and under an incident photon irradiance (400–700 nm) of 100 µmol photons m^−2^ s^−1^ and 200 µmol photons m^−2^ s^−1^. The dashed lines indicate replicate measurements (three technical replicates per polyp), while the continuous lines indicate LOESS-smoothed trends with 95% confidence intervals (gray shading). Zero depth represents the level of the coral mouth opening, while negative depth values indicate positions inside the GVC.

**Fig 3:**
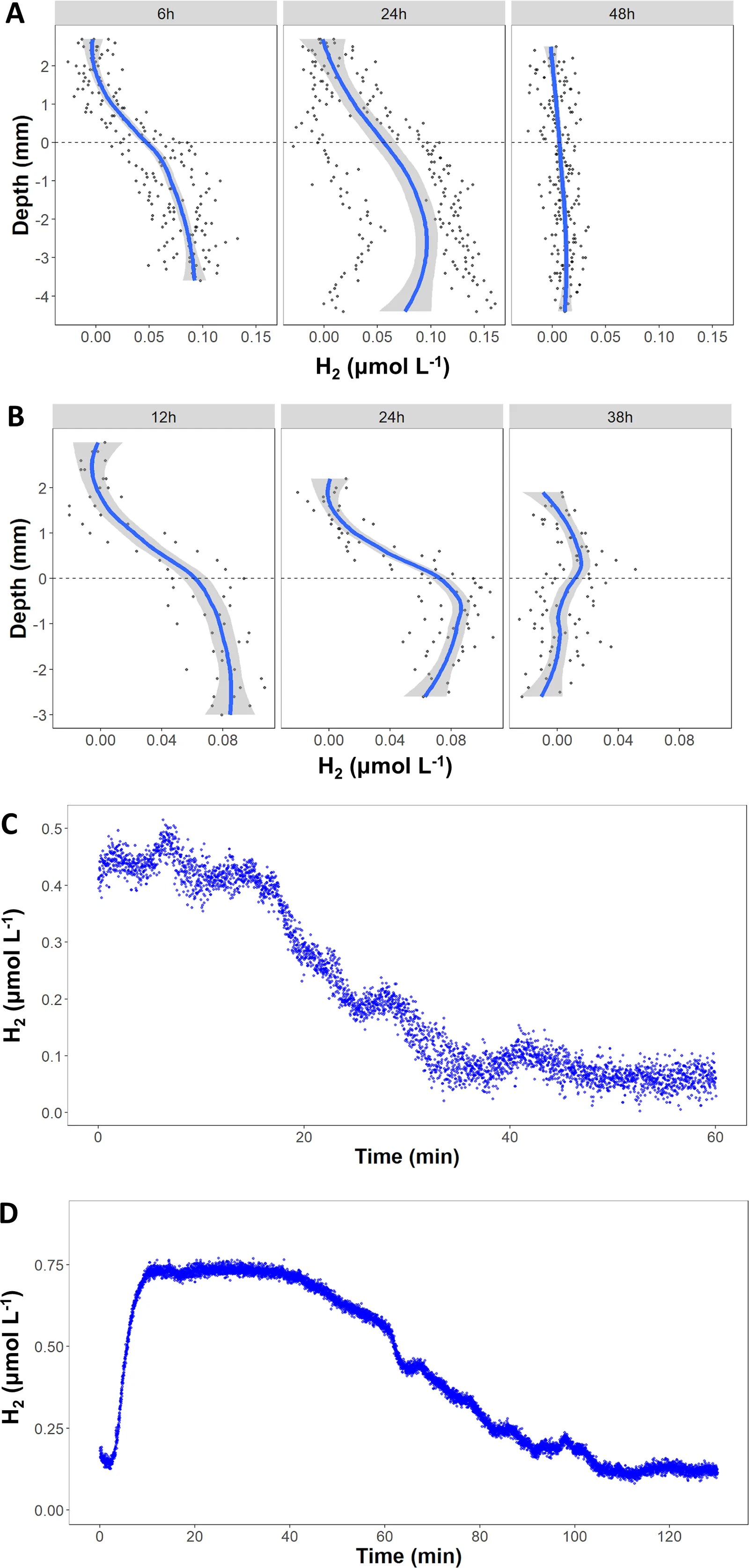
Temporal dynamics of H_2_ concentration in the gastrovascular cavity after feeding under dark conditions. (**A–B**) H_2_ depth profile in the healthy *A. curvata* polyps (*n* = 2, with three technical replicates per polyp). (**C–D**) Continuous measurement in *Goniastrea* sp.; the microsensor tip was positioned at 50% of polyp depth. (**C**) Measurement started after H_2_ concentration stabilized; (**D**) measurement started after light off.

Despite low pH and hypoxic/anoxic conditions in the GVC of dark-incubated, healthy, or bleached *A. curvata* corals, we did not detect any H₂S, which would be indicative of active sulfate reduction in the GVC. Instead, we documented pronounced H₂ dynamics in the GVC of dark- and light-incubated corals (Fig. 2 and 3). In healthy polyps, average H_2_ concentrations were low under dark (0.22 ± 0.34 µmol H_2_ L^−1^, Table S2) and low light (0.27 ± 0.24 µmol H_2_ L^−1^) conditions, but decreased to near-zero under high light (0.00 ± 0.01 µmol H_2_ L^−1^), with significantly lower values compared to dark and low light treatments (one-way ANOVA, F_(2, 6)_ = 18.11, *P* < 0.01; Tukey HSD, *P* < 0.05). In contrast, bleached polyps exhibited consistently detectable H_2_ concentrations across all light conditions, with no significant differences among treatments.

We also monitored the H_2_ concentration dynamics in the GVC after food ingestion. In *A. curvata* polyps, the H_2_ concentration remained elevated (0.10 μmol H_2_ L^−1^ and 0.08 μmol H_2_ L^−1^ in replicates 1 and 2, respectively) for at least 24 h, before returning to zero at 36–48 h (Fig. 3A and B). In *Goniastrea* sp. polyps collected from the field, the continuous measurements revealed rapid changes in H_2_ concentration following feeding (Fig. 3C and D). H_2_ level increased to 0.5 μmol H_2_ L^−1^ in replicate 1 (Fig. 3C); in replicate 2, the H_2_ concentration increased to 0.75 μmol H_2_ L^−1^ within 10 min after the light was switched off and began to decrease after 1 h (Fig. 3D).

**Fig 4:**
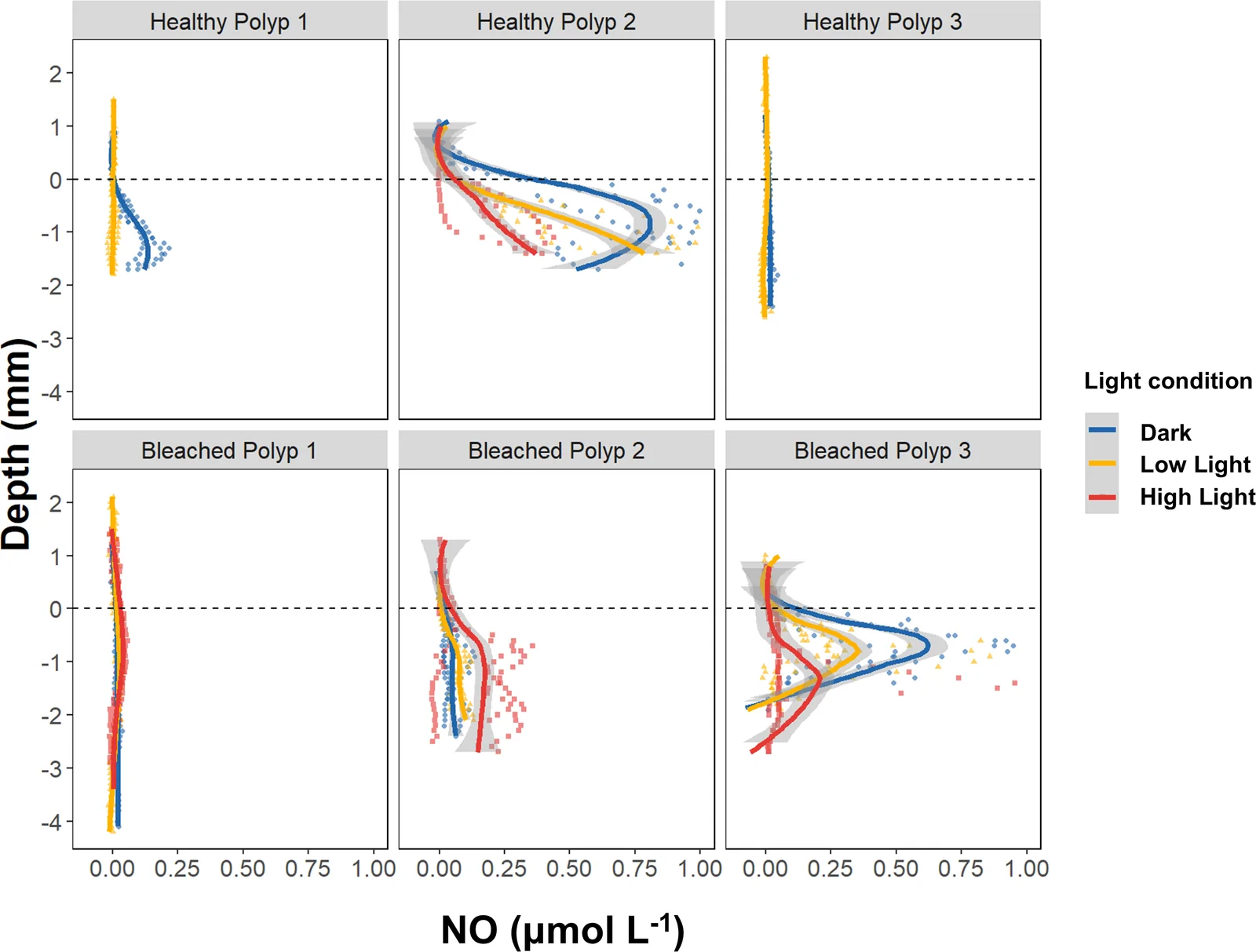
Depth profiles of NO in the gastrovascular cavity of healthy and bleached *A. curvata* polyps in darkness and under an incident photon irradiance (400–700 nm) of 100 µmol photons m^−2^ s^−1^ and 200 µmol photons m^−2^ s^−1^. The dashed lines indicate replicate measurements (three technical replicates per polyp), while the continuous lines indicate LOESS-smoothed trends with 95% confidence intervals (gray shading). Zero depth represents the level of the coral mouth opening, while negative depth values indicate positions inside the GVC.

**Fig 5:**
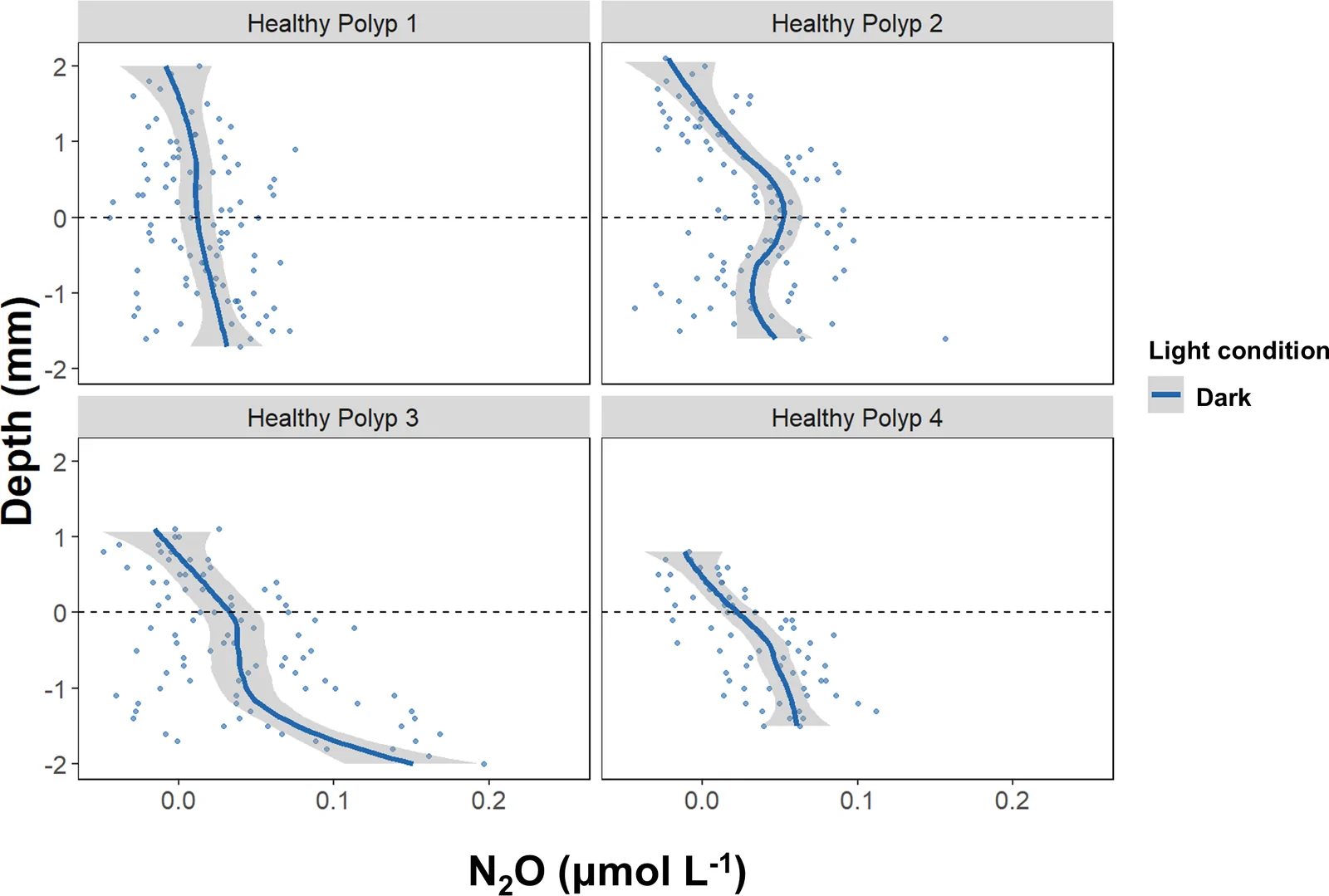
Depth profiles of N_2_O in the gastrovascular cavity of healthy *A. curvata* polyps under dark conditions. These measurements were conducted on a separate set of healthy *A. curvata* polyps from a different colony than those used for the other microsensor measurements. The dashed lines indicate replicate measurements (three technical replicates per polyp), while the continuous lines indicate LOESS-smoothed trends with 95% confidence intervals (gray shading). Zero depth represents the level of the coral mouth opening, while negative depth values indicate positions inside the GVC.

The intermediate products of the denitrification pathway, NO and N_2_O, were also detected under hypoxic conditions in the GVC of *A. curvata*. Some samples exhibited very high NO concentrations, reaching up to 1.2 μmol NO L^−1^ in Healthy Polyp 2 and 0.95 μmol NO L^−1^ in Bleached Polyp 3 (Fig. 4). In healthy corals, NO followed the same light-dependent pattern as H_2_, decreasing with increasing irradiance: the average NO concentration decreased from 0.28 μmol NO L^−1^ in the dark to 0.06 μmol NO L^−1^ under high light. Compared with the other polyps, Healthy Polyp 3 and Bleached Polyp 1 exhibited consistently low NO concentrations, remaining below 0.06 μmol NO L^−1^ across all light conditions. We observed high variability in NO dynamics among technical replicates. For example, under high light conditions: in Bleached Polyp 2, two technical replicates exceeded 0.3 μmol NO L^−1^, whereas one remained below 0.05 μmol NO L^−1^; in Bleached Polyp 3, two replicates were lower than 0.06 μmol NO L^−1^, while one reached as high as 0.95 μmol NO L^−1^. In contrast, the N_2_O concentrations remained relatively stable in the GVC, ranging from 0.08 to 0.20 μmol N_2_O L^−1^ in healthy polyps in the dark (Fig. 5).

### Microbial diversity in the coral GVC

**Fig 6:**
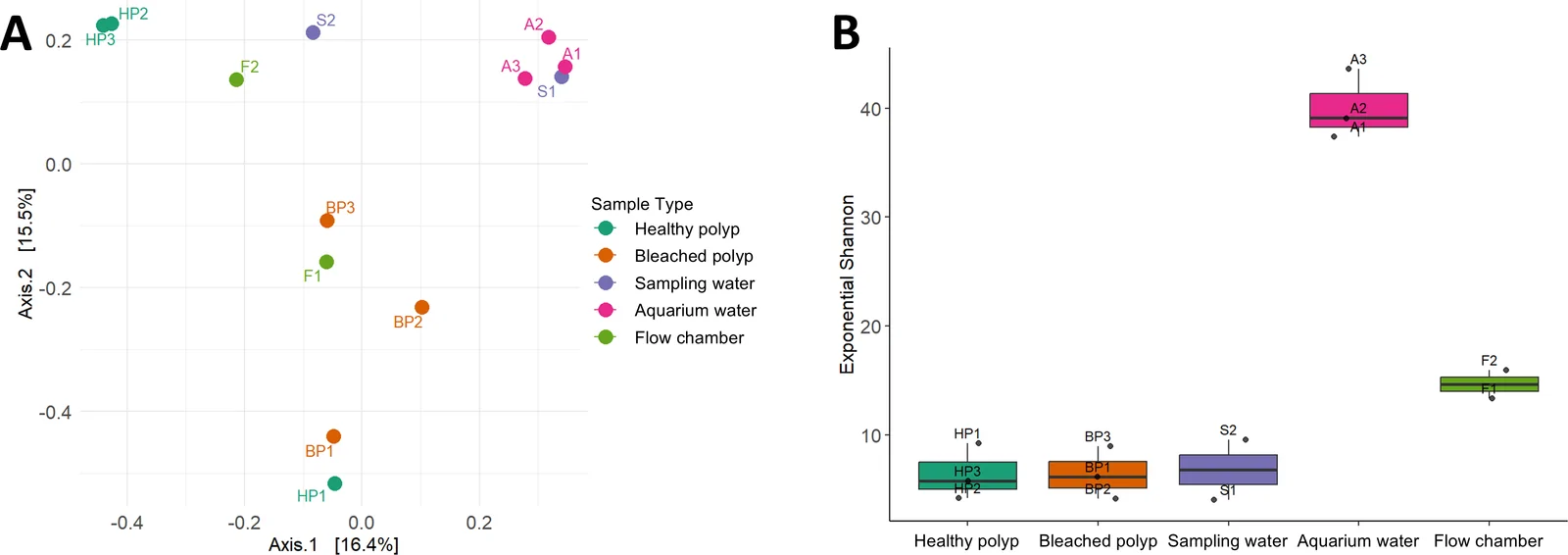
Microbial diversity in the gastrovascular fluid of healthy and bleached *A. curvata* polyps and in environmental samples. (**A**) PCoA based on Bray–Curtis dissimilarities showing differences in bacterial community composition among sample types. (**B**) Alpha diversity represented as the exponential of the Shannon index (e^H^).

Microvolume sampling and subsequent DNA extraction and amplicon sequencing were successful in all sampled polyps of *A. curvata*, yielding (1.2–6.3) × 10^5^ decontaminated reads in GVC samples, aquarium samples, and flow chamber samples, and (2.1–3.5) × 10^4^ reads in filtered seawater samples. PCoA based on Bray–Curtis dissimilarities revealed clustering patterns among five sampling groups (Fig. 6A), which were further supported by relative abundance profiles of bacterial families (Fig. S2). GVC-associated samples (healthy and bleached polyps) generally clustered separately from environmental samples, and healthy and bleached polyps were mostly distinct. However, one healthy polyp sample (Healthy Polyp 1) was positioned closer to the bleached cluster, suggesting some overlap in community composition. PERMANOVA confirmed that microbial community composition differed significantly between groups (*adonis2*: F = 1.25, R^2^ = 0.384, *P* = 0.046). Multivariate dispersion analysis indicated no significant differences in within-group variability (*betadisper*: F = 0.85, *P* = 0.594), confirming that the observed PERMANOVA result was not driven by heterogeneous dispersion.

Alpha diversity, assessed as Shannon’s H (exponential), was broadly comparable among healthy polyps, bleached polyps, sampling water, and flow chamber samples, while aquarium water samples exhibited higher diversity (Fig. 6B). However, Kruskal–Wallis testing did not detect significant differences across groups (χ^2^ = 9.08, *P* = 0.059), and pairwise Wilcoxon rank-sum tests with Benjamini–Hochberg correction did not reveal significant contrasts (all adjusted *P* > 0.33). These results indicate that overall alpha diversity is largely comparable across different sampling groups, reflecting similar microbial richness and evenness.

**Fig 7:**
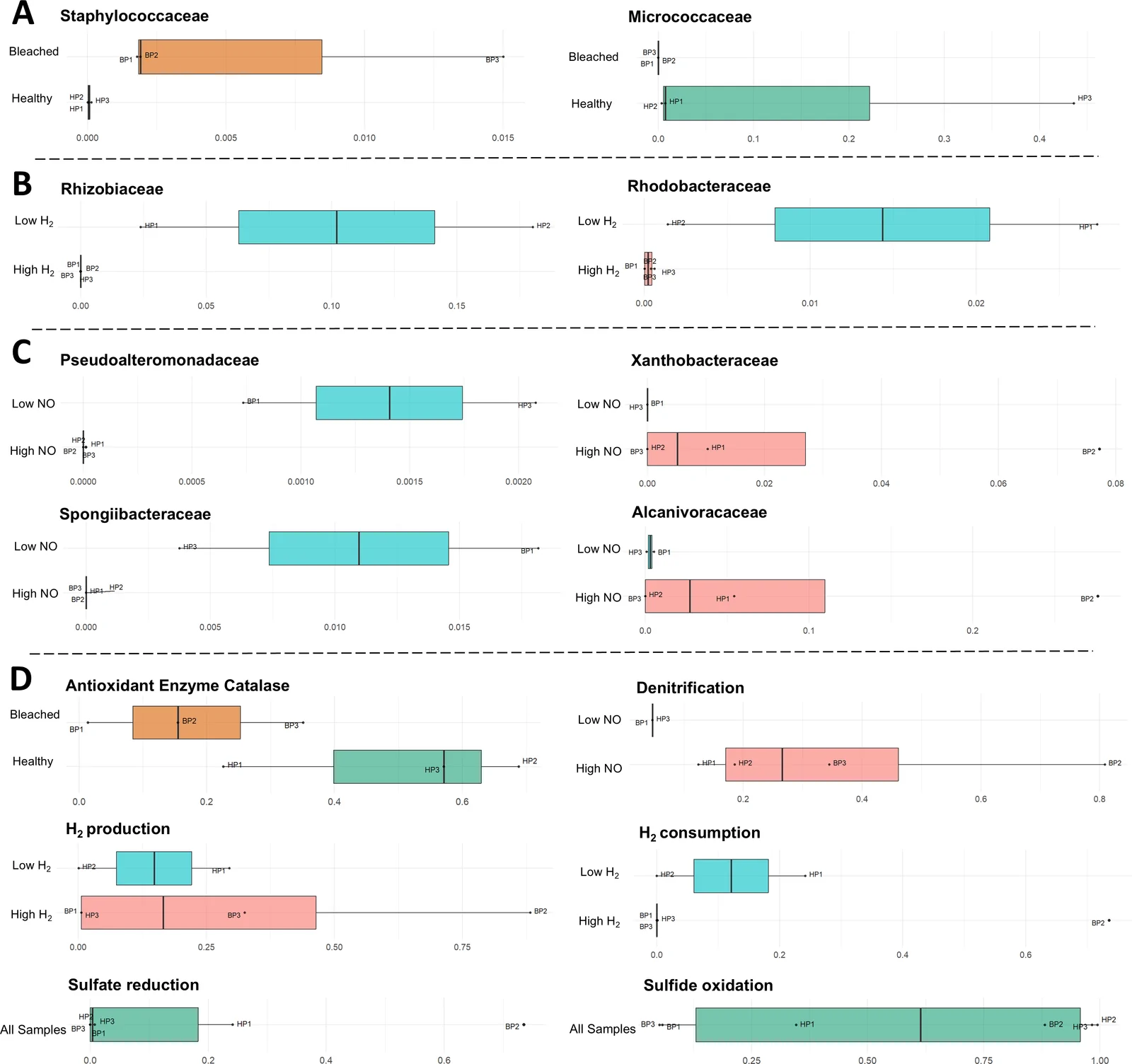
Relative abundance of bacterial families and predicted functions across different sample groups in *Astraeosmilia curvata*. (A–C) Family-level relative abundance of bacterial taxa showing significant differences between groups (*n* = 3): (**A**) healthy or bleached coral polyps; (**B**) high or low GVC H₂ concentration; (**C**) high or low GVC NO concentration. (**D**) Relative abundance of predicted microbial functions based on KEGG Orthologs corresponding to target pathways. Colors indicate sample categories: healthy polyps (green) and bleached polyps (orange); low H_2_ or NO concentrations in the GVC are shown in blue and high concentrations in pink.

To identify the taxa contributing to the observed differences in community composition, we examined the relative abundances of bacterial families between healthy and bleached coral polyps (Fig. 7A). Bleached polyps showed a higher relative abundance of Staphylococcaceae (0.61% ± 1.05% vs 0.13% ± 0.22% in healthy polyps), whereas healthy polyps were enriched in Micrococcaceae (14.9% ± 24.9% vs 0.00% ± 0.00% in bleached polyps). Given the variation in NO and H_2_ concentrations within the GVC among individual coral polyps, we further tested whether these microenvironmental gradients corresponded to shifts in family-level bacterial composition (Fig. 7B and C). Polyps with low H_2_ concentrations exhibited higher abundances of Rhizobiaceae (10.21% ± 11.04% vs 0.0% ± 0.0% in high-H_2_ polyps) and Rhodobacteraceae (1.44% ± 1.83% vs 0.03% ± 0.03%). Polyps with low NO concentrations were enriched in Pseudoalteromonadaceae (0.07% ± 0.01% vs 0.00% ± 0.00% in high-NO polyps) and Spongiibacteraceae (0.55% ± 0.86% vs 0.00% ± 0.00%), whereas Xanthobacteraceae (0.52% ± 0.73% vs 1.93% ± 3.97%) and Alcanivoracaceae (2.71% ± 3.84% vs 7.06% ± 13.72%) were more abundant in high-NO polyps.

We next evaluated the functional profiles of the GVC-associated microbiomes using predicted gene abundances, focusing on antioxidant activity, denitrification, hydrogen, and sulfur metabolism (Fig. 7D). Importantly, we note that these predictions are based on the taxonomic profiles present in each sample and therefore do not represent the true presence or absence of metabolic pathways or genes of interest. The predicted abundance of the antioxidant enzyme catalase appeared to be higher in healthy polyps compared with bleached polyps (49.5% ± 24.0% and 17.3% ± 16.9%, respectively), although this difference was not statistically significant (*P* = 0.20). For denitrification-related genes, polyps with low NO concentrations exhibited lower predicted abundances (<5%) than those with high NO concentrations (12.5%–80.8%), but the difference did not reach statistical significance (*P* = 0.13). Given the limited number of biological replicates (*n* = 3 per group), these comparisons have limited statistical power and should be interpreted cautiously. Predicted functions related to H_2_ production and consumption were comparable among groups, with no statistically significant differences detected. The *nifH* gene (associated with nitrogen fixation) showed a predicted relative abundance of 3.2% in Bleached Polyp 2, mostly attributed to Xanthobacteraceae, while its abundance was below 0.1% in all other samples. We found relatively high abundances of predicted sulfate-reducing genes in Healthy Polyp 1 and Bleached Polyp 2, accounting for 24.2% and 73.1% of the community, respectively, whereas all other polyps showed levels below 1%. Predicted sulfide-oxidizing genes were generally more prevalent, reaching 5%–6% in Bleached Polyps 1 and 3, 34.6% in Healthy Polyp 1, and as high as 95.3% ± 6.2% in the other polyps.

## DISCUSSION

Our microsensor measurements reveal that the coral GVC is a highly dynamic chemical microenvironment that is affected by different microbial processes. Combined with taxonomic analysis of the GVC-associated microbiome, our findings highlight the tight coupling of host physiology, symbiont photosynthesis, and microbial metabolism in shaping microenvironmental conditions within coral polyps.

### Persistent hypoxia and anaerobic metabolism in bleached coral GVC

The “high light” and “low light” levels (200 and 100 μmol photons m^−2^ s^−1^, respectively) were chosen relative to the aquarium environment (150 μmol photons m^−2^ s^−1^) and are far lower than the high light levels experienced in natural reef habitats. Nevertheless, despite the relatively low light levels, pronounced diel dynamics in O_2_ and pH were observed in healthy polyps. From light to dark, the GVC of healthy polyps shifted from hyperoxic to hypoxic, with a substantial drop in pH. In contrast, O_2_ and pH profiles in the GVC of bleached coral polyps consistently exhibited hypoxic and acidic conditions under dark and different light conditions, reflecting the loss of O_2_ production by Symbiodiniaceae. These altered chemical conditions between bleached and healthy polyps likely reshape the balance between aerobic and anaerobic metabolism within the holobiont. In bleached polyps, the production of H_2_ and NO persisted under illumination, indicating that anaerobic processes, such as fermentation and denitrification, are sustained across the diel cycle. This persistence suggests a shift in energy and nutrient cycling pathways in the bleached coral holobiont, which may in turn be accompanied by changes in microbial community structure. However, because bleaching in the present study occurred during transport rather than through a controlled bleaching induction protocol, the observed microbiome differences should be interpreted with caution and cannot be attributed solely to bleaching, as they may also reflect transport-related stress or other environmental changes. In bleached polyps, we observed a marked decline in aerobic bacteria (Micrococcaceae). In contrast, the high predicted abundance of the antioxidant enzyme catalase in two out of three healthy polyps suggests that microbes associated with healthy corals may possess greater potential for ROS detoxification in response to photosynthesis-associated oxidative stress, although this prediction requires validation through direct functional measurements.

### Bleaching-associated shifts in coral microbiome composition

Beyond the pronounced changes in the GVC chemical microenvironment between healthy and bleached polyps, the collapse of the coral-algae symbiosis also induces other changes in host physiology that significantly influence the associated microbiome. Microbial community restructuring during bleaching has been widely reported (35–41). This restructuring likely arises from shifts in energy metabolism following symbiont loss and from a reduction in host immune function that reduces the host’s ability to regulate its microbiome (42). Although different coral species vary in the stability or flexibility of their bacterial community structure (37, 43), a common pattern across studies is a shift toward higher abundances of heterotrophic and potentially pathogenic bacteria in bleached corals (36, 41). We observed a higher relative abundance of Staphylococcaceae in the GVC of bleached polyps. Members of this family include opportunistic pathogens associated with host stress (44–46), which may reflect these physiological changes in the coral host. This shift suggests that the GVC becomes a niche more permissive to opportunistic pathogens, potentially increasing the susceptibility of bleached corals to subsequent disease (47). Environmental stress can also directly shape microbial communities, e.g., through changes in temperature or elevated nutrients (43, 48). However, in this study, we did not induce bleaching under controlled experimental conditions, so we cannot disentangle the direct effects of environmental variation.

### Microbial mediation of carbon and nitrogen cycling

The detection of H_2_, NO, and N_2_O provides direct evidence of active anaerobic microbial processes within the GVC. These pathways represent key components of carbon and nitrogen turnover in both environmental and host-associated microbial communities. Fermentative H_2_ production reflects microbial degradation of organic carbon and generates organic acids and alcohols that can fuel downstream anaerobic metabolisms (49). In principle, hydrogen gas production could also indicate N_2_ fixation, where H_2_ can be a byproduct (50). Denitrification reduces nitrate and nitrite to gaseous nitrogen species (NO, N_2_O, and N_2_), thereby removing bioavailable nitrogen from the holobiont and helping to maintain the C–N balance, which is crucial for the stability of the coral-algae symbiosis (51–54). These anaerobic pathways are shaped not only by oxygen availability but also by the local concentrations of organic carbon and inorganic nitrogen substrates (55). Because the GVC is the primary site of food acquisition and digestion, feeding directly enriches the cavity with organic matter, providing substrates that support fermentation and denitrification. Previous studies of the coral GVC identified bacterial taxa with known sulfate reduction and sulfide oxidation potential (5, 24), a finding in line with our functional predictions of sulfate-reduction and sulfide-oxidation genes in several coral polyps. While we did not directly detect H_2_S in the GVC, active sulfur cycling may still occur within the GVC microenvironment, e.g., due to close spatial association between sulfate-reducing and sulfide-oxidizing bacteria and/or efficient chemical sulfide oxidation in the GVC. Together, these observations indicate that the GVC functions as an important site of microbial activity, which may play a major role in the internal nutrient cycling of the coral holobiont.

### Effects of heterotrophic feeding on GVC microenvironment

Our H_2_ measurements after food ingestion reflect dynamic changes in the availability of organic matter within the GVC. In *A. curvata* polyps, our observation showed that H_2_ concentrations declined to undetectable levels after 36–48 h, indicating low levels of residual organic substrates. In addition to declining substrate availability, the decrease in H_2_ concentrations may also reflect active microbial consumption within the GVC, as molecular hydrogen is a potent energy and electron source utilized by diverse microorganisms. Several detected taxa, including members of Xanthobacteraceae, Rhodobacteraceae, and Rhizobiaceae, possess hydrogen-oxidizing capabilities, suggesting that hydrogenotrophic metabolism likely contributes to H_2_ concentration dynamics in the GVC. In *Goniastrea* polyps, measurements started immediately after food ingestion, and H_2_ concentrations rose rapidly within 10 min. This sharp increase suggests a rapid accumulation of labile organic carbon in the GVC following food ingestion, driven by coral digestive processes and microbial decomposition. As microbial carbon metabolism is triggered by the availability of organic carbon (56), we speculate that nitrogen cycling is similarly influenced by nitrogen-containing organic compounds and exhibits feeding-induced dynamics, as observed, e.g., in the vertebrate gut (57–59). In this study, we performed a single artificial feeding. However, under natural conditions, microbial activity in the GVC likely fluctuates with feeding frequency, food composition, digestive efficiency, and the retention time of food residues, leading to strong temporal variability in both carbon turnover and nitrogen transformations.

### Linking chemical gradients to microbial community structure

When comparing the GVC microbial communities of polyps with consistently low NO concentrations (i.e., Healthy Polyp 2 and Bleached Polyp 1) with other polyps, we found that no taxa with predicted denitrification genes were detected in the low-NO group, while taxa with denitrification potential were detected in every high-NO sample. Additionally, several bacterial families showed clear shifts in relative abundance. High-NO polyps were enriched in Xanthobacteraceae, a family that includes many denitrifiers and has been reported to perform partial denitrification (60, 61), and Alcanivoracaceae, which also possess denitrification potential (62). Consistently, our PICRUSt2 analysis identified ASVs assigned to Alcanivoracaceae that were predicted to encode nitrite reductase, nitric oxide reductase, and nitrous oxide reductase, suggesting a potential role for this family in nitrogen oxide transformations within the GVC. In the low-NO polyps, Pseudoalteromonadaceae were more abundant. This family is characterized by strong protease production and organic nitrogen degradation (63), and only a few species within this family are capable of denitrification (64). We found that the relative abundance of predicted *nifH* genes in our coral polyps was generally low. Previous studies have shown that the abundance and activity of diazotrophic microorganisms in corals can vary substantially depending on host species, trophic strategy, and environmental conditions (65–67). Our results therefore suggest a potentially limited role for diazotrophy in *A. curvata* maintained long-term in aquaria. Consequently, diazotrophy in the GVC may represent a context-dependent process influenced by host physiology and nutrient availability.

In bleached corals, the loss of symbiotic algae reduces the primary energy supply for the host, which consequently has to rely more on heterotrophic feeding and changes in the composition of ingested food (14–16). Increased coral heterotrophic feeding may enhance microbial activity in the GVC. However, in this study, we did not quantitatively control the amount of food provided or the coral polyp size, and therefore cannot directly compare substrate concentrations to infer different reaction rates. In addition, NO and N_2_O are intermediate products of denitrification and do not fully represent the overall process; applying the acetylene inhibition technique, which blocks the conversion of N_2_O to N_2_, before N_2_O measurements would provide a more accurate quantification of denitrification activity (68, 69).

We note that variability among technical replicates, particularly for nitric oxide measurements, was relatively high. This variability likely reflects strong microscale heterogeneity within the coral gastrovascular cavity, where chemical gradients can change rapidly over small spatial scales. However, the limited number of technical and biological replicates makes it difficult to fully distinguish the biological variability from other sources of variation. The small number of biological replicates (*n* = 3 per group) also limits statistical power, and non-significant results should therefore be interpreted cautiously rather than as evidence for the absence of biological differences. Furthermore, the use of colonies that bleached during transport rather than through a controlled stress regime does not allow us to disentangle the specific effects of bleaching from other potential stressors. Future studies incorporating larger sample sizes, multiple species, and experimentally induced bleaching will help to further clarify the dynamics of anaerobic microbial processes within coral GVC microenvironments.

Our study focused on taxonomic composition and taxonomy-based functional prediction in the GVC microbiome. While these predictions can contribute to meaningful hypothesis generation, they do not necessarily reflect the actual presence, expression, or activity of functional genes. For example, although sulfate-reduction pathways were predicted, we did not detect measurable H_2_S concentrations within the GVC. This may indicate a decoupling between genetic potential and *in situ* activity, influenced by local environmental constraints, such as oxygen availability, substrate limitation, or the rapid removal of H_2_S by sulfur-oxidizing bacteria. However, a simpler explanation is that while these taxa are close relatives of sulfate-reducing bacteria, they do not necessarily possess genetic potential for sulfate reduction themselves. A metagenomic or metatranscriptomic approach is required for true functional profiling. Future studies that incorporate controlled feeding experiments, measurements of carbon and nitrogen contents in the GVC, in concert with metabolic gene profiling, will be essential for resolving the role of the GVC-associated microbiome in holobiont C–N cycling.

In summary, this study demonstrates that the coral gastrovascular cavity is a chemically dynamic microenvironment and a hotspot of microbial activity. Based on microsensor measurements of H_2_, NO, and N_2_O, we show that the GVC supports active anaerobic microbial processes, such as fermentation and denitrification, and we also identify corresponding microbial taxa and predicted functional genes within the GVC microbiome. In healthy corals, these anaerobic activities are suppressed under illumination by the hyperoxic conditions generated through symbiont photosynthesis. In contrast, the GVC of bleached corals remained consistently hypoxic and acidic, allowing anaerobic metabolic processes to persist across the diel cycle. Such patterns indicate a shift in energy and nutrient cycling pathways within the bleached coral holobiont. Furthermore, feeding experiments confirmed that the availability of organic substrates is an important driver of H_2_ dynamics, indicating that coral feeding behavior can strongly influence microbial activity within the GVC. Collectively, these findings establish the coral GVC as a critical site of microbial activity that is closely linked to host physiology and stress status. Future quantitative studies integrating microbial, physiological, and biogeochemical perspectives will be essential for resolving its contributions to internal nutrient cycling and to holobiont functioning under environmental change.

## Data Availability

Raw sequencing data have been deposited in the NCBI Sequence Read Archive (SRA) under BioProject accession number PRJNA1417609. The microsensor data generated in this study have been deposited in the Zenodo repository and are publicly available at https://doi.org/10.5281/zenodo.21342726.

## SUPPLEMENTAL MATERIAL

Supplemental material aem.02441-25-s0001.docx Tables S1 and S2; Fig. S1 and S2.

ASM does not own the copyrights to Supplemental Material that may be linked to, or accessed through, an article. The authors have granted ASM a non-exclusive, world-wide license to publish the Supplemental Material files. Please contact the corresponding author directly for reuse.
