# Supplementary material for: Heterotrophic feeding and symbiotic state shape the chemical microenvironment, microbiome composition, and activity in the coral gastrovascular cavity
Source: Appl Environ Microbiol. 2026 Jul 27;92(8):e02441-25. doi: 10.1128/aem.02441-25 (PMC13488293; doi:10.1128/aem.02441-25)
Supplement: Supplementary file 1 [file aem.02441-25-s0001.docx]

**SUPPLEMENTARY INFORMATION**

**Table S1.** Microbial functions and corresponding KEGG Orthology (KO) IDs.

| Function | Enzyme | KEGG Orthology (KO) ID |
| --- | --- | --- |
| Antioxidant enzyme catalase | catalase | ko:K03781 |
| Denitrification |  | ko:K00370, ko:K00371, ko:K00374, ko:K02567, ko:K02568, ko:K00368, ko:K15864, ko:K04561, ko:K02305, ko:K00376 |
| Nitrogen fixation | nifH | ko:K02588 |
| H_2_ production | ferredoxin hydrogenase (EC 1.12.7.2), | ko:K00532, ko:K00533, ko:K00534, ko:K06441, ko:K18016, ko:K18017, ko:K18023 |
|  | iron hydrogenase (EC:1.12.7.-) | ko:K25123, ko:K25124 |
|  | bifurcating [FeFe] hydrogenase (EC 1.12.1.4) | ko:K17997, ko:K17998, ko:K17999 |
|  | [NiFe] hydrogenase | ko:K18008, ko:K00437 |
|  | formate hydrogenlyase | ko:K15827, ko:K15828, ko:K15829, ko:K15830, ko:K15831, ko:K15832, ko:K22015 |
|  | hydrogenase (EC 1.12.99.6) | ko:K06281, ko:K06282, ko:K23548, ko:K23549 |
|  | hydrogenase (EC 1.12.1.2) | ko:K00436, ko:K18005, ko:K18006, ko:K18007 |
|  | hydrogenase (EC 1.12.1.3) | ko:K17992, ko:K17993, ko:K17994, ko:K18330, ko:K18331, ko:K18332 |
|  | nitrogenase (EC 1.18.6.1) | ko:K00531, ko:K02586, ko:K02588, ko:K02591 |
|  | vanadium-dependent nitrogenase (1.18.6.2) | ko:K22896, ko:K22897, ko:K22898, ko:K22899 |
| H_2_ consumption | quinone-reactive Ni/Fe-hydrogenase (EC:1.12.5.1) | ko:K05922, ko:K05927 |
|  | acetogenesis | ko:K00192, ko:K00195, ko:K00196, ko:K00198 |
|  | sulfhydrogenase (EC:1.12.1.3, 1.12.1.5) | ko:K17993, ko:K17994, ko:K17995, ko:K17996 |
| Sulfate reduction |  | ko:K11180, ko:K11181, ko:K27196, ko:K27187, ko:K27188, ko:K27189, ko:K27190, ko:K27191 |
| Sulfide Oxidation |  | ko:K17222, ko:K17223, ko:K17224, ko:K17225, ko:K17226, ko:K17227, ko:K22622, ko:K17218, ko:K21307, ko:K21308, ko:K21309 |

**Table S2** Mean concentrations (±SD) and statistical analysis of microsensor profiles in the GVC of *Caulastrea curvata* (corresponding to Figures 1–4).

Microsensor data were averaged over the portion of each profile with depths ≤ 0, and mean values and standard deviations were then calculated across different light conditions, coral symbiotic states, and time points following food ingestion (n = 3). Superscripts indicate statistical significance: ***** and ****** denote comparisons between healthy and bleached polyps, and letters (a, b, c) denote comparisons across light conditions and time points following food ingestion.

|  | | Dark | Low light | High light |
| --- | --- | --- | --- | --- |
| O_2_  (μmol L^-1^) | Healthy polyps | 41.29 ± 23.99 ^a^**^*^** | 167.00 ± 35.26 ^b^**^*^** | 271.08 ± 56.15 ^c^**^*^** |
|  | Bleached polyps | 77.51 ± 24.38 ^a^**^*^** | 111.82 ± 32.99 ^a,b^**^*^** | 145.95 ± 23.14 ^b^**^**^** |
| pH | Healthy polyps | 7.33 ± 0.17 ^a^**^*^** | 7.81 ± 0.03 ^b^**^*^** | 7.89 ± 0.13 ^b^**^*^** |
|  | Bleached polyps | 7.55 ± 0.08 ^a^**^*^** | 7.54 ± 0.10 ^a^**^**^** | 7.56 ± 0.18 ^a^**^**^** |
| H_2_  (μmol L^-1^) | Healthy polyps | 0.22 ± 0.34 ^a^**^*^** | 0.27 ± 0.24 ^a^**^*^** | 0.00 ± 0.01 ^b^**^*^** |
|  | Bleached polyps | 0.40 ± 0.21 ^a^**^*^** | 0.15 ± 0.27 ^a^**^*^** | 0.08 ± 0.01 ^a^**^**^** |
| NO  (μmol L^-1^) | Healthy polyps | 0.28 ± 0.41 ^a^**^*^** | 0.15 ± 0.27 ^a^**^*^** | 0.06 ± 0.12 ^a^**^*^** |
|  | Bleached polyps | 0.15 ± 0.20 ^a^**^*^** | 0.09 ± 0.09 ^a^**^*^** | 0.08 ± 0.06 ^a^**^*^** |
| H_2_ conc. after food ingestion in the dark | Time | 6h | 24h | 48h |
|  | H_2_ (μmol L^-1^) | 0.08 ± 0.02 ^a^ | 0.09 ± 0.06 ^a^ | 0.01 ± 0.00 ^a^ |
|  | Time | 12h | 24h | 38h |
|  | H_2_ (μmol L^-1^) | 0.08 ± 0.02 ^a^ | 0.08 ± 0.00 ^a^ | 0.00 ± 0.02 ^b^ |

Figure S1 Rarefaction curves showing the relationship between sequencing depth and number of ASVs for each sample.

Figure S2 Relative abundance of bacterial families in the gastrovascular fluid of healthy and bleached *Caulastrea* *curvata* polyps and in environmental samples. Taxa with relative abundances below 1% across all samples were grouped as “Other”.
